# The Screening and Isolation of Ethyl-Carbamate-Degrading Strains from Fermented Grains and Their Application in the Degradation of Ethyl Carbamate in Chinese Baijiu

**DOI:** 10.3390/foods12152843

**Published:** 2023-07-27

**Authors:** Siyu Xue, Naihui Dong, Kexin Xiong, Hui Guo, Yiwei Dai, Huipeng Liang, Yingxi Chen, Xinping Lin, Beiwei Zhu, Sufang Zhang

**Affiliations:** SKL of Marine Food Processing & Safety Control, National Engineering Research Center of Seafood, School of Food Science and Technology, Dalian Polytechnic University, Dalian 116034, China; morganxue@126.com (S.X.); 15942416365@163.com (N.D.); xiongkx1953@163.com (K.X.); 21173083200047@xy.dlpu.edu.cn (H.G.); daiyiwei@dlpu.edu.cn (Y.D.); lhpdxx@126.com (H.L.); chenyx@dlpu.edu.cn (Y.C.); yingchaer@163.com (X.L.);

**Keywords:** ethyl carbamate (EC), *Candida ethanolica*, Chinese Baijiu, immobilization

## Abstract

Ethyl carbamate (EC), a 2A carcinogen produced during the fermentation of foods and beverages, primarily occurs in distilled spirits. Currently, most studies focus on strategies for EC mitigation. In the present research, we aimed to screen strains that can degrade EC directly. Here, we report two *Candida ethanolica* strains (J1 and J116), isolated from fermented grains, which can reduce EC concentrations directly. These two yeasts were grown using EC as the sole carbon source, and they grew well on different carbon sources. Notably, after immobilization with chitosan, the two strains degraded EC in Chinese Baijiu by 42.27% and 27.91% in 24 h (from 253.03 ± 9.89 to 146.07 ± 1.67 and 182.42 ± 5.05 μg/L, respectively), which was better than the performance of the non-immobilized strains. Furthermore, the volatile organic compound content, investigated using gas chromatography-mass spectrometry, did not affect the main flavor substances in Chinese Baijiu. Thus, the yeasts J1 and J116 may be potentially used for the treatment and commercialization of Chinese Baijiu.

## 1. Introduction

Ethyl carbamate, also referred to as EC and urethane, is a detrimental substance generated during the processing and fermentation of foods and beverages. It is considered a processing contaminant as it has been classified as a 2A carcinogen and is probably carcinogenic to humans [1,2,3]. Studies have shown that EC can yield a vinyl carbamate epoxide in the human body. When bound to DNA as adducts, EC can increase the possibility of mutations and carcinogenesis. The hazardous properties of EC have spurred efforts [4] to find ways of mitigating its formation or enhancing its degradation during processing [5]. Notably, a previous study on co-exposure to EC and ethanol indicated that the interaction between these two compounds contributed to EC-mediated tumorigenesis [6].

EC formation routes during the fermentation of food and beverage mainly include two pathways: the microbial metabolism of arginine into EC and the reaction of ethanol with cyanogenic glycosides to produce EC [7]. Hence, the ongoing strategies for reducing the EC concentration incorporate both inhibition of the precursors (arginine and cyanogenic glycosides) of EC formation and direct degradation of EC. Compared to *Schizosaccharomyces pombe*, *Wickerhamomyces anomalus* is able to inhibit the precursors of EC via metabolism [8,9]. A commercial strain, ECMo01, can degrade urea and reduce EC concentrations by 50% and 90% in bread and red wine, respectively [10]. During fermentation, after *Saccharomyces cerevisiae* ZJU replaced the traditional fermentation yeast, the EC concentrations in yellow rice wine were reduced by 90%, while the effect on flavor substances and the volatile organic compounds (VOCs) were not investigated [11,12]. Urease, generally recognized as safe, can successfully reduce mostly urea [13]. However, the effect of elimination of the EC precursor, urea, on fermented food has not been discussed in detail. Deletion of *CAR1* (coding arginase in *S. cerevisiae*) using CRISPR-Cas9 has been demonstrated to reduce EC concentrations by 70% [14,15,16,17]. However, in microorganisms, EC precursors are produced in various ways; hence, multiple genes have to be suppressed to reduce EC synthesis [18]. Methods of directly degrading EC have also been proposed, such as the use of urethanase and microorganisms possessing degradation activity similar to urethanase [12,19]. EC hydrolase from *Acinetobacter calcoaceticus* can decrease the EC concentration in liquor by 71.60 µg/L in 12 h [20]. *Lysinibacillus sphaericus* MT33 can degrade up to 60% of the EC in wine [21]. Therefore, screening strains that can degrade EC is a promising approach for reducing EC concentrations.

In a report on alcoholic beverages from the Food and Drug Administration, the EC concentration in most samples was less than 100 µg/L, while that in sherry wine exceeded 500 µg/L [22,23]. Furthermore, distilled spirits typically contain more EC than wine and beer [24,25,26]. Many countries have established regulations and recommendations for limiting EC in alcoholic beverages; for example, in Canada, the permitted EC concentration in Sake is less than 200 µg/L, while it is less than 150 µg/L in distilled spirits; in USA, the permitted limit for EC concentration in distilled spirits is less than 125 µg/L; and in Europe, the EC limitation in distilled spirits from the Czech Republic and France is less than 150 µg/L [27]. Chinese Baijiu, one of the world’s oldest and most popular distilled spirits, contains high levels of EC, which have to be mitigated.

In the present study, we aimed to reduce the EC content in fermented distilled spirits. Toward this, we screened strains from fermented grains that can utilize EC as a carbon source for growth and verified their ability to degrade EC directly in Chinese Baijiu. Simultaneously, as the high-concentration ethanol and acidic environment in Chinese Baijiu limits the strains’ ability to degrade EC, we used chitosan to immobilize the strains and compared the effect of immobilization on the EC content in Chinese Baijiu. Furthermore, changes in the VOC content of the Chinese Baijiu were also detected before and after treatment with the immobilized strains. The strains screened in the present study will be conducive to further research regarding their mechanism of action and the commercialization of Chinese Baijiu.

## 2. Materials and Methods

### 2.1. Materials

Ultrapure water was produced by the TW-D24UV system of Millitrack (Haryana, India). The yeast nitrogen base without amino acids (YNB), yeast extract peptone dextrose (YPD), chitosan, and chloramphenicol were purchased from Sangon Biotech (Shanghai, China). Chinese Baijiu (52%, *v*/*v*) was purchased from a supermarket (Dalian, China). EC was procured from Sigma-Aldrich Co. (St. Louis, MO, USA). TES buffer (300 mmol/L NaCl, 50 mmol/L Tris-HCl, 25 mmol/L EDTA, 0.2% (*v*/*v*) SDS, 2 mg/mL proteinase K, pH 8.0) was purchased from TransGen Biotech (Beijing, China). Tris-saturated phenol solution (pH 8.0), absolute ethanol, sodium acetate (pH 5.3), CaCl_2_, boric acid, and chloroform-isoamyl alcohol were purchased from Sangon Biotech (Shanghai, China). Glucose, glycerol, sucrose, mannose, and sorbose were purchased from Damao Co. (Tianjin, China). 4-methyl-1-pentanol was purchased from Aladdin Biotech (Shanghai, China).

### 2.2. Sample Collection

The fermented grain samples were collected from a vinegar factory in Shandong Province, China. The sample was collected and stored at 4 °C immediately to preserve the microbial consortia until further use [28].

### 2.3. Screening of EC-Degrading Isolates

Approximately 5 g of the fermented grain sample was added to 45 mL of physiological saline and agitated on a shaker at 28 °C for 24 h. The supernatant was then aseptically serially diluted to 10^−7^ dilution. The isolates were grown on nutrient agar plates using the spread plate method. Briefly, 10 µL of the dilutions was spread over the YNB solid medium (YNB 6.70 g, agar 20 g, and sterile ultrapure water 1 L) with 10 mg/L EC as the sole carbon source. These solid media were incubated for 96 h until an independent colony was isolated.

A single colony from the plate was resuspended in 10 μL physiological saline for microscopic examination and a photograph was captured to observe the morphology. Only yeast, identified using microscopic examination was used in the present study. When the morphology was uniform, individual colonies were picked and repeatedly dilution-streaked until a single colony was obtained. When the morphology of the selected colony was not uniform, another single colony was selected and streaked until a colony with uniform morphology was obtained. The strains were stored at −80 °C with 20% glycerol (final concentration) for further analysis.

### 2.4. Phylogenetic Identification of the Isolates

The method for extraction of the genomic DNA from the isolates is described below. The strains stored at −80 °C were resuspended in 1 mL purified water and centrifuged at 13,000× *g* for 30 s. This was followed by the addition of 400 μL TES buffer (300 mmol/L NaCl, 50 mmol/L Tris-HCl, 25 mmol/L EDTA, 0.2% (*v*/*v*) SDS, 2 mg/mL proteinase K, pH 8.0) and 0.3 g glass beads (0.20–0.40 cm), and then they were incubated at room temperature for 5 min. Next, the sample was incubated on an ice bath for 1 min and agitated on a vortex shaker (maximum power) for 1 min; this procedure was repeated five times. Then, 200 μL Tris-saturated phenol solution (pH 8.0) and 200 μL chloroform-isoamyl alcohol were added and incubated for 5 min at room temperature. This was followed by incubation in an ice bath for 1 min and shaking for 1 min for a total of five times and centrifugation at 13,000× *g* at 4 °C for 10 min. The supernatant was put in a separate tube to which 1/10th volume of 3 mol/L sodium acetate (pH 5.3) and 2 volumes of ice-cold absolute ethanol were added and incubated at −20 °C for 20–60 min. This was again followed by centrifugation at 13,000× *g* at 4 °C for 10 min; the supernatant was discarded, and the precipitate was washed repeatedly with 1 mL of 75% ethanol via inversion and then centrifugation at 13,000× *g* for 2 min. The supernatant was discarded, and the pellet was air-dried. An appropriate amount of TER (1 μL 10 μg/μL RNase in 1 mL TE buffer) was added and incubated at 37 °C for 20 min [29].

The potential isolates were identified using 26S rDNA sequencing. Amplification of the 26S rDNA was performed using Taq polymerase chain reaction (PCR) master mix with the D1/D2 primers for bacteria and fungi, respectively (D1: 5′-GCATATCAATAAGCGGAGGAAAAG-3′; D2: 5′-GGTCCGTGTTTCAAGACGG-3′). The reaction was performed for 30 cycles using the following standard PCR parameters: DNA pre-heating at 94 °C for 3min, denaturation at 95 °C for 15 s, annealing at 50 °C for 15 s, primer extension at 72 °C for 1 min, and elongation at 72 °C for 15 min.

The PCR products were subjected to sequencing and comparison using the basic local alignment search tool (BLAST) program from the National Centre for Biotechnology Information (NCBI). The sequences of the isolated strains were aligned with previously reported sequences of closely related species using the ClustalW multiple sequence alignment tool. Additionally, a phylogenetic tree was created using the neighbor-joining method of MEGA 7.0. The accuracy of the tree was evaluated by performing a bootstrap analysis with 1000 replications. Based on the findings from the phylogenetic tree, several yeast species were chosen for further experiments.

### 2.5. Assimilation of Carbon Sources

In total, six carbon source assimilation media were prepared, which included glucose, glycerol, sucrose, mannose, sorbose, and EC. The isolated and purified yeasts were gradient diluted seven times from 10^8^ CFU/mL to 10^9^ CFU/mL. Thereafter, they were inoculated on medium plates and incubated at 28 °C for 48 h. After taking them out, the growth conditions of each yeast on each medium were compared and the suitable carbon source growth conditions for each yeast were summarized and imaged.

### 2.6. Growth Curve Analysis of the Isolated Strains

Wells containing the prepared YNB medium supplemented with 0 g/L, 5 g/L, and 10 g/L of EC were inoculated with 10^8^ CFU/mL of the isolated strains and incubated at 28 °C. The growth of the isolated strains was measured at 600 nm (OD_600_) using a Bioscreen C analyzer system (Labsystems, Helsinki, Finland) [30]. Each microplate well containing 300 μL medium (YNB with 0 g/L, 5 g/L, and 10 g/L EC) was inoculated with 10 μL inoculum. The microplates were incubated for 48 h at 28 °C, and the OD_600_ of each microplate well was monitored hourly. The microplates were shaken for 10 s before each measurement. Three replicate wells for each condition were performed in each assay.

### 2.7. Degradation of EC in Chinese Baijiu by the Immobilized Strain

The strain was immobilized according to a previously reported method [31]. Briefly, 10^9^ CFU of the strain was added dropwise into 100 mL hardening liquid (0.6% CaCl_2_ and 5% boric acid, *w*/*v*) and incubated for 5 h. Then, the beads were dropped into 2% (*w*/*v*) chitosan solution and incubated for 40 min. After filtration, the beads were divided into four parts. For each part, beads were filtered and added every four hours, at 4 h, 8 h, 12 h, and 24 h. The samples were extracted according to a previously reported method [21]. Briefly, a 2 mL sample was prepared at 4 h, 8 h, 12 h, and 24 h. The strains that were not immobilized and the beads without strains were used as the control groups, and the sample was prepared at 24 h. The EC concentration was assayed using gas chromatography-mass spectrometry (GC-MS) [32]. The oven program was as follows: held at 50 °C for 1 min, ramped up to 180 °C at 8 °C/min, and then to 210 °C at 15 °C/min and held at 210 °C for another 5 min. Subsequently, the samples (2 µL) were injected for GC/MS analysis.

### 2.8. Determination of VOC Content in Chinese Liquor

Chinese Baijiu (100 mL) was treated with 100 mg beads with immobilized J1 and immobilized J116 and beads without strains at room temperature for 24 h. The VOCs in Chinese liquor were identified according to a previously reported method [33]. The Chinese Baijiu was placed in a headspace sampling bottle with 6 mg/L 4-methyl-1-pentanol as the internal standard. The bottle was then incubated at 60 °C for 25 min. After incubation, an SPME fiber (Supelco, Inc., Bellefonte, PA, USA) was inserted into the headspace to extract VOCs for 25 min. The fiber was then extracted and placed into the GC-MS 7890A/5977A (Agilent Technologies Inc., Palo Alto, CA, USA) equipped with a flexible capillary vessel column (HP-INNOWAC, 30 m × 0.25 mm, 0.25 µm film thickness) for VOC analysis. The temperature of the oven was programmed as follows: starting at 35 °C for 5 min and then increased at a rate of 3 °C per minute up to 50 °C and maintained for 3 min; subsequently raised to 150 °C at a rate of 4 °C per minute; further increased to 250 °C at a rate of 20 °C per minute; and then held for 5 min. The mass spectra were obtained using an ionization potential of 70 eV. The mass scan range covered 15 to 300 m/z. The injector was operated in splitless mode with a flow rate of 1 mL/min. The NIST 14 spectrum library was used to compare the compounds. Analysis of VOCs was performed by comparing the GC peak area with that of the internal standard.

### 2.9. Statistical Analysis

All experiments were performed in triplicate, and the results are shown as the mean ± standard deviation. The statistical analyses were performed using SPSS version 19.0 (SPSS Inc., Chicago, IL, USA). The normality of the distribution of the data population (Shapiro–Wilk test) and the homogeneity of variance in the samples (Levene’s test) were tested. And all data in the results show normal distribution. Subsequently, one-way analysis of variance (ANOVA) with Duncan’s test was used to determine statistical differences, and differences were considered significant at *p* < 0.05.

## 3. Results and Discussion

### 3.1. Isolation and Screening of EC-Degrading Strains from Fermented Grains

In total, 138 isolates grew on YNB solid medium with 10 mg/L EC as the sole carbon source (Figure S1). After repeated streaking on YPD agar medium to obtain the purest culture, these strains were observed under a microscope. Some strains were found to be bacteria and were not included in this study. Therefore, after the morphology analysis, 75 yeasts were selected for subsequent experiments. The results of the specific morphology analysis are shown in Figure S2.

**Figure 1:**
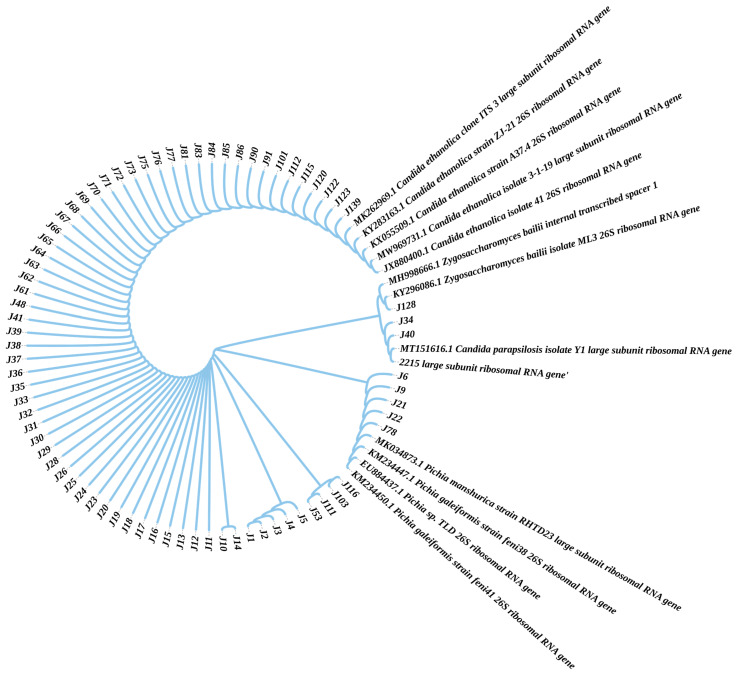
The phylogenetic trees of the strains that can grow with EC as the carbon source. In these strains, J6, J9, J21, J22, and J78 were identified as *Pichia manshurica*, *Pichia galeiformis,* and *Pichia* sp. Strain J34 and J40 exhibited the most homology to *Candida parapsilosis* isolate Y1 and *Candida parapsilosis culture* CBS:2215, respectively. Strain J128 exhibited the most homology to *Zygosaccharomyces bailii* isolate ML3. And the remaining isolates exhibited the most homology to *Candida ethanolica* isolate ZJ-2*1*, *Candida ethanolica* isolate A37.4, *Candida ethanolica* isolate 41, and *Candida ethanolica* isolate 3-1-19. Blue arrows show the strains (J1, J10, J14, J34, J53, J78, J116, J128) selected for further research.

26S rRNA sequencing was used to characterize these strains. The phylogenetic trees of these strains were constructed. Based on the results of strain identification shown in Figure 1, strains J6, J9, J21, J22, and J78 were identified as *Pichia manshurica*, *Pichia galeiformis*, and *Pichia* sp. Strains J34 and J40 exhibited maximum homology to *Candida parapsilosis* isolate Y1 and *Candida parapsilosis* culture CBS:2215, respectively. Strain J128 exhibited maximum homology to *Zygosaccharomyces bailii* isolate ML3. The remaining isolates (the details are shown in Figure 1) exhibited maximum homology to *Candida ethanolica* isolate ZJ-21, *Candida ethanolica* isolate A37.4, *Candida ethanolica* isolate 41, and *Candida ethanolica* isolate 3-1-19. Hence, based on the results of the phylogenetic tree, eight strains (J1, J10, J14, J34, J53, J78, J116, and J128) were selected for subsequent experiments.

### 3.2. Carbon Source Assimilation by the Isolated Strains

Carbon sources exert diverse effects on the growth of yeast. In this study, glucose, glycerin, sucrose, mannitol, sorbitol, and EC were used as the carbon source test materials. The eight yeast strains (J1, J10, J14, J34, J53, J78, J116, and J128) were selected for the carbon source assimilation experiment and their ability to use different carbon sources is shown in Table 1. All strains grew in medium containing these carbon sources, with the exception of J53, which could not grow in the medium with glycerin. Strains J1, J10, J14, and J116, identified as *Candida ethanolica*, grew well in the presence of glucose, glycerin, and sorbitol. Mannitol could be the optimal carbon source for J1 and J10. Notably, the growth condition of J1 and J116 on EC was better than that of the other strains. Strain J34, identified as *Candida parapsilosis*, grew in the presence of all these carbon sources, especially in the medium with glycerin. Although strain J78 could grow in the presence of all these carbon sources, the overall carbon source assimilation capacity was not good. Strain J78, identified as *Pichia* sp., was not selected for further experiments. Strain J128, identified as *Zygosaccharomyces bailii*, utilized all these carbon sources and grew well in medium containing glycerin. Hence, strains J1, J10, J14, J34, J116, and J128 were selected for the growth curve analysis. A study has shown that yeast extracted from corncob preferentially uses glucose, followed by xylose and arabinose [34], while it can efficiently use glycerin to produce arabitol with high selectivity, and that the volumetric productivity improved from 0.13 to 0.33 g/L-h [35]. Therefore, carbon sources may also selectively determine the production of metabolites. The carbon source assimilation ability is an indicator of the ability of microorganisms to use different carbon sources and a physiological and biochemical indicator for microbial identification, which will be useful for identifying the auxiliary characteristics of microorganisms. Furthermore, the ability to utilize carbon sources determines whether yeast can become a chassis cell in the future.

### 3.3. Growth Curve of the Isolated Strains

**Figure 2:**
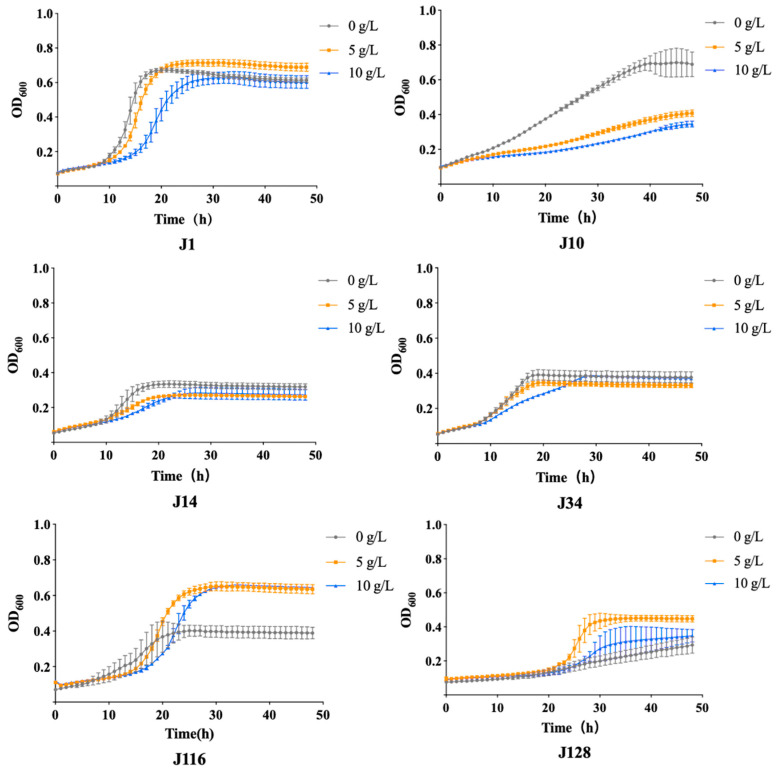
Growth curve of isolated strains of J1, J10, J14, J34, J116, and J128. The growth curves were measured for 48 h. The concentration of these mediums was prepared with 0, 5, and 10 g/L. Gray lines present 0 g/L, yellow lines present 5 g/L, and blue lines present 10 g/L.

In this study, EC was used as the sole carbon source for J1, J10, J14, J34, J116, and J128. As shown in Figure 2, according to the OD_600_ value, J1 and J116 showed strong vigor in the presence of different concentrations of EC, while J10, J14, J34, and J128 grew poorly. Regarding J10, the OD_600_ of the control group (0.64) was better than that when EC (5 g/L and 10 g/L) was used as the carbon source (0.38 and 0.34, respectively). Thus, J10 was not the ideal strain for EC degradation. For strains J14 and J34, the OD_600_ did not vary significantly (around 0.32 and 0.38, respectively) after treatment with no carbon source and EC as the carbon source for 48 h. J128 did not grow well. The logarithmic growth period and lag period were not obvious, and the OD_600_ value was not high (only 0.43). Yeast J128 grew better in 5 g/L EC medium than in 10 g/L EC medium. This is supposedly because J128 could use EC as a carbon source; however, 10 g/L EC affected the growth of J1, because of which, the strain could not proliferate.

The OD_600_ values of J1 and J116 indicated the existence of three different phases, namely lag, propagation, and stationary phases. We observed that in a certain time range, the fermentation of strain J1 in different media increased with the fermentation time, indicating that the growth of each strain in the media was good and that it was the best time for these strains to utilize EC. In the medium without EC, OD_600_ started to reduce due to the scarcity of carbon sources. In the medium containing 5 g/L and 10 g/L EC, the logarithmic growth period was prolonged to 25 h and 32 h, respectively. In the stationary phase, the OD_600_ value (0.73) in the final 5 g/L medium was higher than that in the 10 g/L medium (0.68). As shown in Figure 2, J116 in the free-carbon group entered the propagation phase after 8 h of inoculation and entered the stationary phase after 27 h, which was followed by the senescence phase after 27 h. The lack of carbon sources did not affect the growth of the strain, and the OD_600_ value reached 0.42. In the presence of 10 g/L EC, the logarithmic growth period of J116 was slightly delayed compared to that observed in the 5 g/L medium, although the final OD_600_ value was almost the same (0.63). Furthermore, in the medium lacking other carbon sources, the OD_600_ value of J1 was better than that of J116, indicating that the EC utilization capacity of J1 was better than J116. Hence, J1 and J116 were selected for detecting EC degradation and changes in flavor substance composition.

### 3.4. Degradation of EC in Chinese Wine by Strains and Immobilized Strains

**Figure 3:**
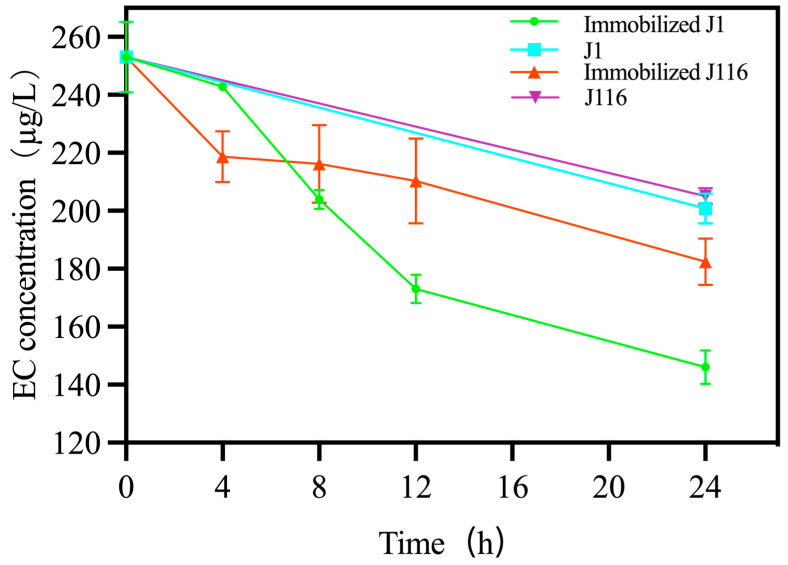
The EC concentration of Chinese Baijiu after treatment with immobilized J1, J1, and immobilized J116, J116 for 24 h. (The error bar shows the SD).

As the high-concentration ethanol and acidic environment in Chinese Baijiu limits the EC degradation ability of yeast, we used yeasts and immobilized yeasts to degrade the EC in Chinese Baijiu. As shown in Figure 3 and Table S1, the EC concentration decreased significantly by 24 h in these systems. Unlike the non-immobilized strains, the EC degradation ability of the chitosan-immobilized J1 and J116 improved significantly. In particular, the immobilized J1 decreased the EC concertation from 253.03 ± 9.89 μg/L to 146.07 ± 1.67 μg/L, while the non-immobilized J1 decreased it from 253.03 ± 9.89 μg/L to 200.80 ± 4.17 μg/L. Similarly, the immobilized J116 decreased the EC concentration from 253.03 ± 9.89 μg/L to 182.42 ± 5.05 μg/L, while the non-immobilized J116 decreased it from 253.03 ± 9.89 μg/L to 205.11 ± 2.29 μg/L. The immobilized J1 degraded EC by 106.96 μg/L (42.27%), which was more than that of J116 (70.61 μg/L by 27.91%), while non-immobilized J1 and J116 degraded only 52.23 μg/L (20.64%) and 47.92 μg/L (18.93%), respectively. After immobilization, the degradation rate of J1 was twice that of the non-immobilized strain, while the degradation rate of immobilized J116 was 1.47 times that of the non-immobilized strain. Therefore, the immobilized strain was a convenient and effective tool for removing EC from Chinese Baijiu; furthermore, the beads can be removed easily, which is conducive to the treatment and subsequent commercialization of Chinese Baijiu.

Next, we compared the EC degradation rate in each sampling interval. Collectively, immobilized J1 and J116 significantly decreased the EC concentration by 42.27% and 27.91% in 24 h, respectively. With immobilized J1, the EC concentration decreased by 4.01% in 0–4 h, 15.41% in 4–8 h, 13.17% in 8–12 h, and 9.68% in 12–24 h. With immobilized J116, the EC concentration decreased by 13.59% in 0–4 h, 1.00% in 4–8 h, 2.27% in 8–12 h, and 11.05% in 12–24 h. In the first four hours, the degradation rate of J116 was faster than that of J1, which slowed down thereafter. The rate of EC degradation by J1 was rapid between 4–12 h. In a previous study, *Lysinibacillus sphaericus* MT33 decreased the EC content in rice wine by 153.04 µg/L in 3 days, which was considerably longer than that observed (24 h) in the present study [21]. Strikingly, the reduction in EC content by J1 is nearly twice that obtained with immobilized *Rhodotorula mucilaginosa* (54.18 µg/L) in 7 days. However, the ability of strains J1 and J116 to degrade EC is not as good as that of purified ethyl carbamate hydrolase (65.50% in 12 h) [20]. Therefore, the specific mechanism underlying J1- and J116-mediated degradation of EC requires further investigation. In the fermentation of yellow wine using *Saccharomyces cerevisiae* with *Lactobacillus fermentum* as the initial fermentation materials, EC and urea content decreased by 77.71% and 94.16%, respectively [36]. As a fermentation starter culture for distilled spirits, *Lactobacillus brevis* could degrade EC concentrations by 40% compared to the control group without a starter culture [37]. These two studies were adjusted from the perspective of the fermentation strains in order to reduce EC concentration, while our study directly reduced the EC concentration from the perspective of finished Chinese Baijiu. The result of immobilized J1 was similar to *Lactobacillus brevis*, but slightly worse than *Saccharomyces cerevisiae* with *Lactobacillus fermentum* as the initial fermentation material. As for the reason J1 and j116 can degrade EC in Chinese Baijiu, we assume that these two yeast strains contain EC hydrolase. In recent years, many studies have found the existence of EC-degrading enzymes, such as amidase found in *Aspergillus oryzae* [38], *Candida parapsilosis* [39], and *Agrobacterium tumefaciens* D3 [40] and esterase found in *lysinibacillus fusiformis* strain sc02 [12] and *Agrobacterium* sp. [20]. As for the specific enzyme types of J1 and J116 degrading EC, as well as the enzymatic properties, we will conduct a more in-depth discussion in the follow-up study.

### 3.5. Effect of the Immobilized Strains on the VOC Content in Chinese Baijiu

Flavor substances, the most critical constituents of wine, appeal to many consumers. In addition to water and ethanol, 2% of Chinese Baijiu contains other ingredients, such as esters, alcohols, organic acids, and aldehydes. Unlike the decisive flavor substances of other distilled liquors around the world, esters, not alcohols, are the main factors influencing the flavor of Chinese Baijiu [41]. As mentioned previously, esters significantly influence the primary flavor and style of liquor [38].

**Figure 4:**
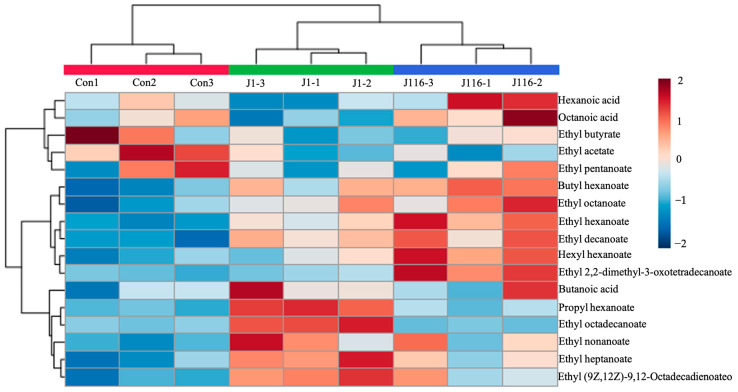
The heatmap of volatile organic compounds in Chinese Baijiu after treatment with immobilized J1, immobilized J116, and at room temperature for 24 h. The heatmap of the 18 esters and 5 organic acids are shown with separated colors on the right side. Red represents the control group. Green represents the immobilized J1 group. Blue represent the immobilized J116 group.

In total, 23 flavor substances, including 18 esters and 5 organic acids, were identified in the commercial Chinese liquor using GC/MS (Table 2 and Figure 4). Ethyl hexanoate (451.94 ± 10.56 mg/L in the control group) was the most abundant flavor substance in both samples, followed by ethyl hexadecanoate (44.98 ± 2.03 mg/L in the control group). The content of ethyl hexanoate exceeded 200 mg/L, indicating that the present Chinese Baijiu was a strongly aromatic liquor [42]. Compared to that in the control group, the concentrations of ethyl acetate, ethyl butyrate, and hexadecyl octanoate in the J1- and J116-treated Chinese Baijiu were reduced. Simultaneously, the contents of ethyl hexanoate, ethyl heptanoate, butyl hexanoate, ethyl octanoate, ethyl nonanoate, hexyl hexanoate, ethyl decanoate, and ethyl (9Z,12Z)-9,12-octadecadienoateo were increased. Notably, in the J1-treated system, the concentration of propyl hexanoate, ethyl 2-phenylacetate, ethyl hexadecanoate, and ethyl (E)-octadec-9-enoate increased significantly. Meanwhile, in the J116-treated system, the concentrations of ethyl 2,2-dimethyl-3-oxotetradecanoate and ethyl hexadecanoate were higher than those in the control group. The concentrations of the remaining flavor substances, especially organic acids, changed slightly (ethyl pentanoate, butanoic acid, hexanoic acid, heptanoic acid, and octanoic acid) (Table 2). Overall, the total contents of the VOCs before and after treatment with immobilized J1 and J116 did not affect the flavor of Chinese Baijiu, as the content of these VOCs exceeded the human threshold values [43]. In other words, the flavor of Chinese Baijiu after treatment with the immobilized strains was not affected significantly. It is noteworthy that the concentration of ethyl hexanoate, the primary flavor substance in Chinese Baijiu, showed an upward trend after treatment under both conditions, while the content of ethyl butyrate remained almost unchanged. Ethyl hexanoate can be generated via two pathways: the synthesis of hexanoic acid and ethanol catalyzed by lipase or esterase and the catalysis of ethanol and acetyl-coenzyme A by ethanol hexanoyl transferase. Based on the stable concentration of hexanoic acid in Chinese Baijiu shown in Table 2, we can infer that the ethyl hexanoate in the J1- and J116-treated Chinese Baijiu was synthesized from acetyl-coenzyme A. However, this hypothesis has to be verified further in the future [44,45,46]. Dong et al. demonstrated that esterase reduces the ester content in Chinese Baijiu [20]. Therefore, we speculated that the enzyme in J1 and J116 might be amidase, rather than an esterase. The strains identified in this study possess the potential to degrade EC effectively and exhibit robust tolerance to high concentrations of ethanol, which can facilitate the industrial and commercial production of Chinese Baijiu. Furthermore, these selected strains may contribute to the development of EC degradation pathways and improve our understanding of the enzymatic mechanisms involved in the degradation of EC. Ultimately, this could lead to the development of novel EC-degrading enzymes and their commercialization for the food and beverage industry.

## 4. Conclusions

In the present study, we aimed to screen strains that can degrade EC directly. As a result, two yeast strains of *Candida ethanolica*, named J1 and J116, were isolated from fermented grains. These two strains were able to grow on 10 g/L EC as the sole carbon source (OD_600_ up to 0.73 and 0.68) and assimilated different carbon sources well. Moreover, the immobilized strain J1 could reduce the EC concentration in Chinese Baijiu from 253.03 ± 9.89 µg/L to 146.06 ± 1.67 µg/L (by 42.27%), while immobilized J116 reduced it to 182.42 ± 5.05 µg/L (by 27.91%) in 24 h. These results were better than those obtained with non-immobilized J1 and J116. Furthermore, the VOC content changed slightly after treatment with immobilized J1 and J116. However, many other factors contributing to EC content reduction still have to be investigated, such as the specific mechanisms via which J1 and J116 reduce EC concentrations in Chinese Baijiu and the causes underlying the changes in VOC content.

## Figures and Tables

**Table 1:** Carbon source assimilation of J1, J10, J14, J34, J53, J78, J116, and J128.

	Glucose	Sucrose	Sorbitol	Glycerin	Mannitol	EC
J1	+++	+	++	+	++	++
J10	+++	+++	+++	+	++	+
J14	+++	+	++	+	+	+
J34	+	+	+	++	+	+
J53	++	+	++	−	++	+
J78	+	+	++	+	+	+
J116	++	+++	++	+	+	++
J128	++	++	++	+	+	+

All strains could grow in the medium with these carbon sources, except for J53 which could not grow in the medium with glycerin. At the same time, J1, J10, J14, J34, J116, and J128 could use carbon sources better than other strains. “−”, “+”, “++”, and “+++” mean that the strains did not grow, did not grow well on the medium, grow well, and grow best, respectively.

**Table 2:** Volatile organic compounds analyzed by HS-SPME/GC-MS in Chinese Baijiu after 24 h.

Compounds	Concentration (mg/L)		
	Control	J1	J116
Ethyl acetate	13.99 ± 1.48 ^a^	9.78 ± 1.24 ^b^	9.74 ± 1.22 ^b^
Ethyl butyrate	31.95 ± 4.49 ^a^	26.03 ± 1.98 ^a^	27.47 ± 2.04 ^a^
Ethyl pentanoate	15.81 ± 1.88 ^a^	14.46 ± 0.81 ^a^	15.15 ± 1.36 ^a^
Ethyl hexanoate	451.94 ± 10.56 ^c^	554.32 ± 15.28 ^b^	638.95 ± 37.72 ^a^
Propyl hexanoate	1.22 ± 0.18 ^b^	5.10 ± 0.27 ^a^	1.86 ± 0.40 ^b^
Ethyl heptanoate	20.30 ± 2.36 ^c^	32.11 ± 1.90 ^a^	26.09 ± 2.22 ^b^
Butyl hexanoate	11.29 ± 1.03 ^b^	15.11 ± 1.23 ^a^	16.91 ± 0.62 ^a^
Ethyl octanoate	46.05 ± 3.09 ^b^	54.54 ± 2.81 ^ab^	57.88 ± 4.01 ^a^
Ethyl nonanoate	1.77 ± 0.18 ^b^	3.63 ± 0.75 ^a^	3.00 ± 0.77 ^ab^
Hexyl hexanoate	23.02 ± 2.25 ^b^	27.45 ± 2.38 ^b^	36.17 ± 2.42 ^a^
Ethyl decanoate	3.77 ± 0.42 ^b^	6.69 ± 0.41 ^a^	7.45 ± 0.96 ^a^
Ethyl 2-phenylacetate	4.86 ± 0.23 ^b^	6.06 ± 0.53 ^a^	5.15 ± 0.51 ^ab^
Ethyl 2,2-dimethyl-3-oxotetradecanoate	8.04 ± 0.48 ^b^	9.29 ± 0.67 ^b^	17.57 ± 1.55 ^a^
Ethyl hexadecanoate	44.98 ± 2.03 ^b^	46.35 ± 2.74 ^b^	62.82 ± 7.32 ^a^
Ethyl octadecanoate	0.63 ± 0.16 ^b^	5.34 ± 0.33 ^a^	0.37 ± 0.12 ^b^
Ethyl (*E*)-octadec-9-enoate	15.71 ± 1.73 ^b^	44.84 ± 1.75 ^a^	14.58 ± 0.93 ^b^
Ethyl (9*Z*,12*Z*)-9,12-Octadecadienoateo	16.42 ± 1.86 ^c^	31.00 ± 1.86 ^a^	24.36 ± 3.65 ^b^
Hexadecyl octanoate	1.59 ± 0.29 ^a^	0.28 ± 0.22 ^b^	0.37 ± 0.03 ^b^
Butanoic acid	2.31 ± 0.19 ^a^	2.74 ± 0.28 ^a^	2.56 ± 0.34 ^a^
Pentanoic acid	1.38 ± 0.21 ^ab^	1.23 ± 0.15 ^b^	1.73 ± 0.18 ^a^
Hexanoic acid	43.33 ± 1.22 ^ab^	39.58 ± 2.00 ^b^	47.21 ± 3.69 ^a^
Heptanoic acid	1.53 ± 0.17 ^a^	1.41 ± 0.21 ^a^	1.81 ± 0.11 ^a^
Octanoic acid	2.51 ± 0.16 ^ab^	2.16 ± 0.12 ^b^	2.77 ± 0.25 ^a^

Different letters in the same column indicate a significant difference (^a,b,c^ *p* < 0.05). Significance only indicates the comparison of the same substance in different groups.

## Data Availability

The data used to support the findings of this study can be made available by the corresponding author upon request.

## Supplementary Materials

The following supporting information can be downloaded at: https://www.mdpi.com/article/10.3390/foods12152843/s1. Figure S1: The strains can grow using EC as the sole carbon source; Figure S2: Results of colony morphological analysis and strain morphological analysis; Table S1: The EC concentration of Chinese Baijiu after treated with immobilized J1, J1, immobilized J116, J116 and chitosan at 4 h, 8 h, 12 h and 24 h.
