# Supplementary material for: The Screening and Isolation of Ethyl-Carbamate-Degrading Strains from Fermented Grains and Their Application in the Degradation of Ethyl Carbamate in Chinese Baijiu
Source: Foods. 2023 Jul 27;12(15):2843. doi: 10.3390/foods12152843 (PMC10416978; doi:10.3390/foods12152843)
Supplement: Supplementary file 1 [file foods-12-02843-s001.zip › foods-2507414-supplementary.pdf]

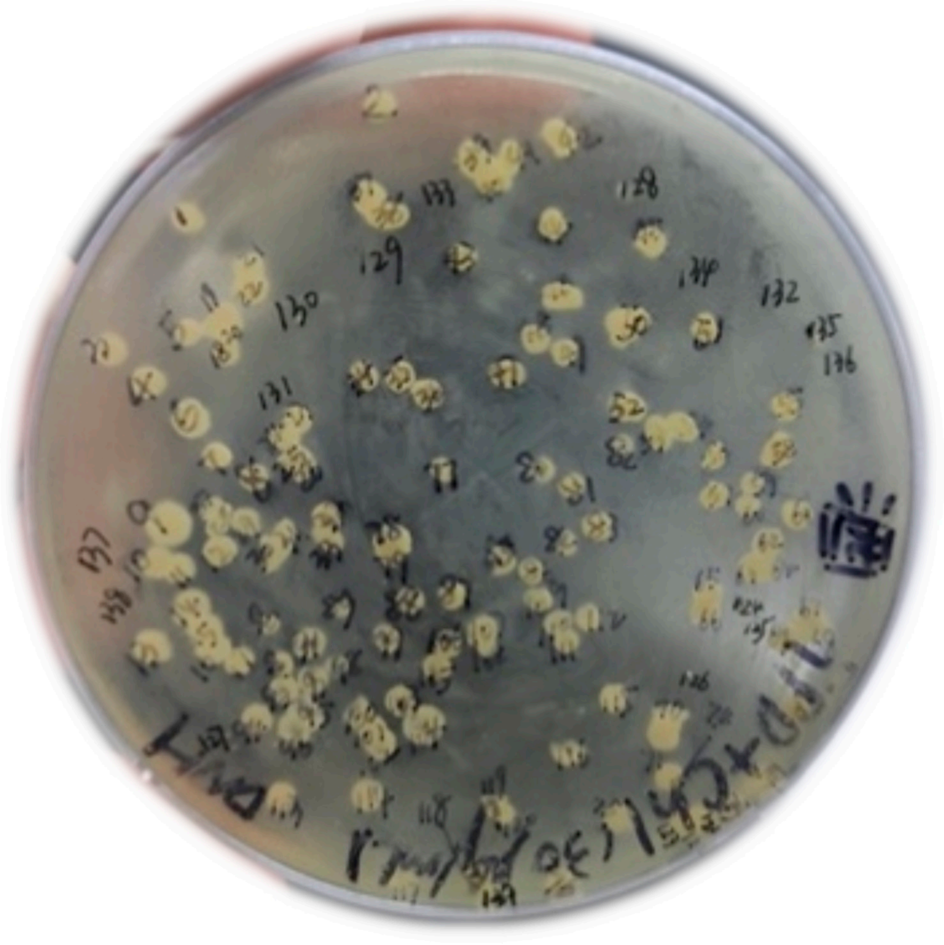

Figure S1 The strains can grow using EC as the sole carbon source.

<table>
<tr><td>Microscopy</td><td>Colony morphology</td><td>Microscopy</td></tr>
<tr><td>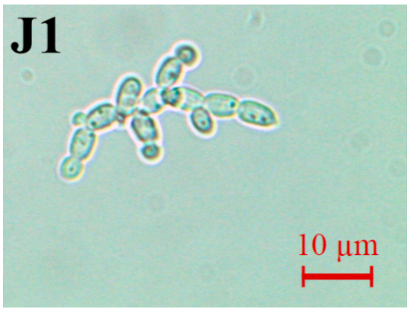
</td><td>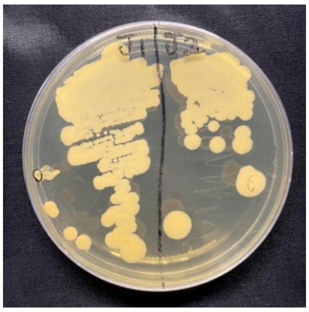
</td><td>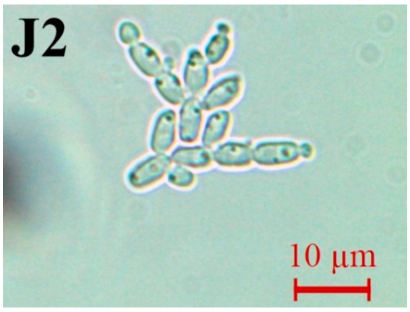
</td></tr>
<tr><td>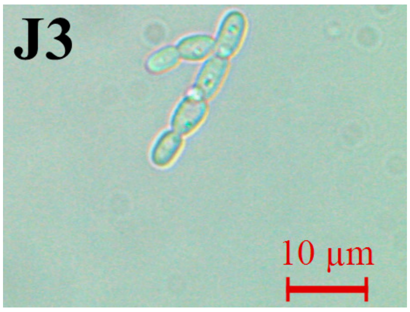
</td><td>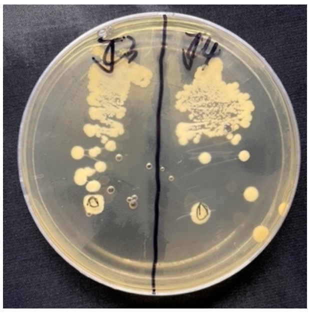
</td><td>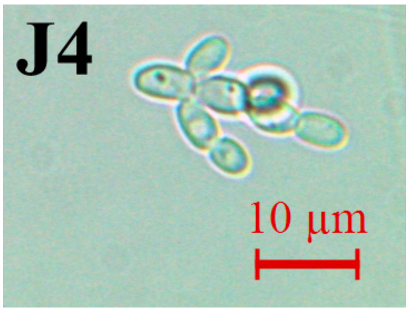
</td></tr>
<tr><td>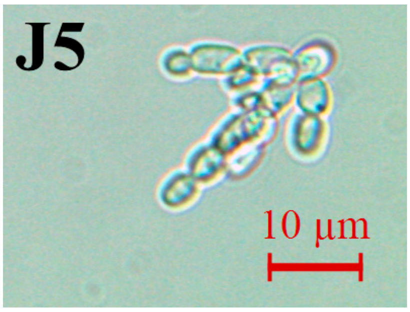
</td><td>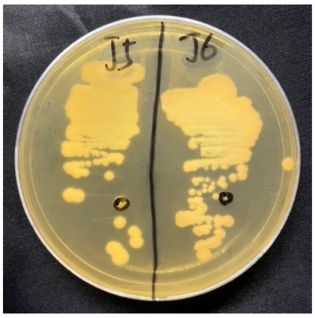
</td><td>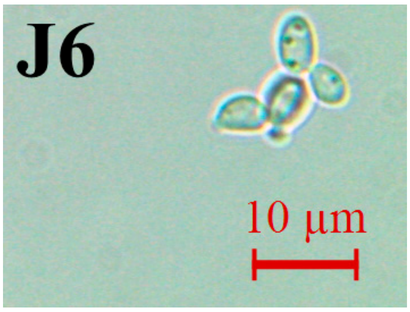
</td></tr>
<tr><td>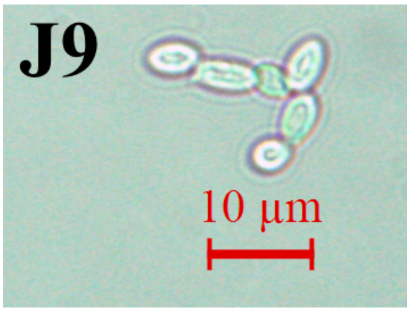
</td><td>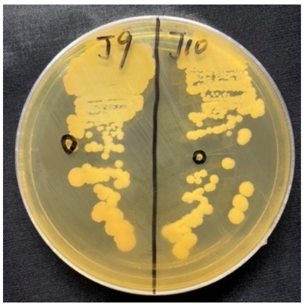
</td><td>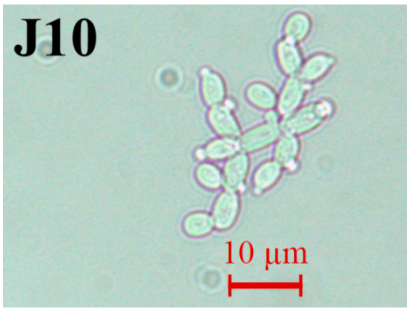
</td></tr>
<tr><td>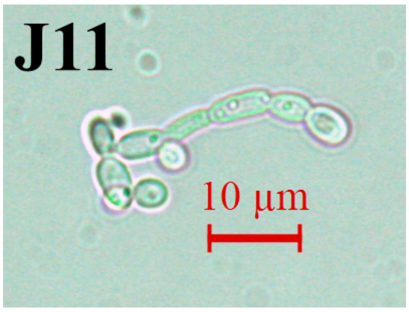
</td><td>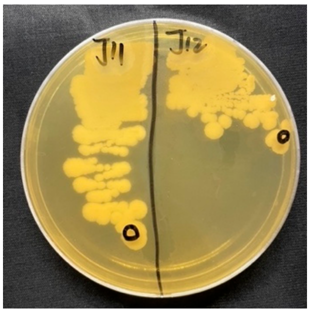
</td><td>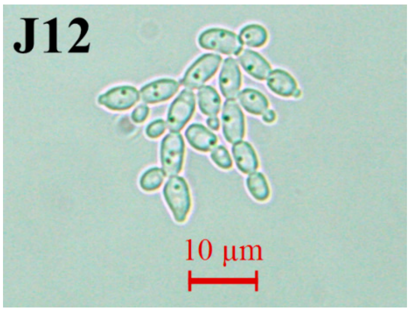
</td></tr>
</table>

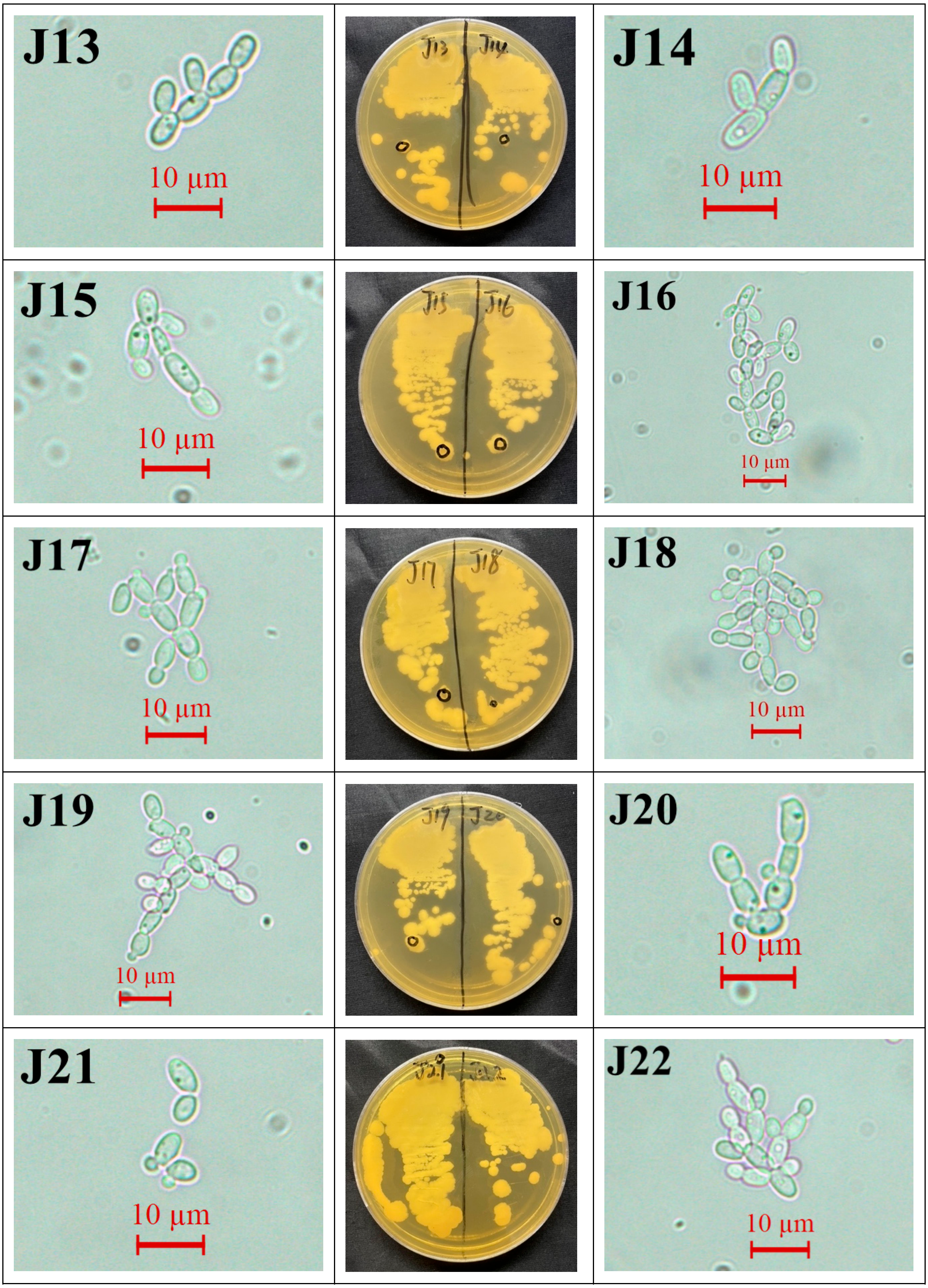
J13
10 µm
J14
10 µm
J15
10 µm
J16
10 µm
J17
10 µm
J18
10 µm
J19
10 µm
J20
10 µm
J21
10 µm
J22
10 µm

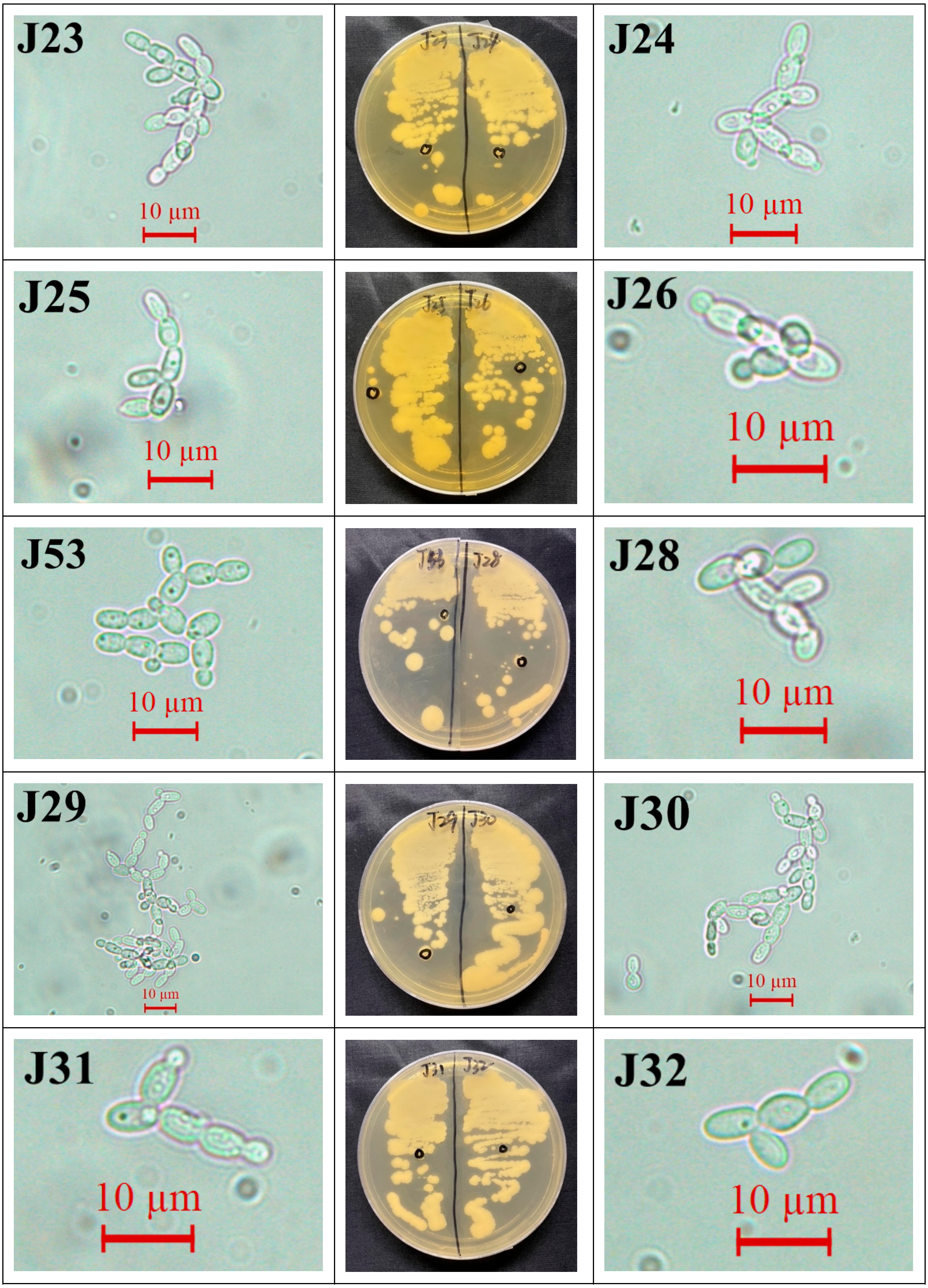
J23
10 µm
J24
10 µm
J25
10 µm
J26
10 µm
J53
10 µm
J28
10 µm
J29
10 µm
J30
10 µm
J31
10 µm
J32
10 µm

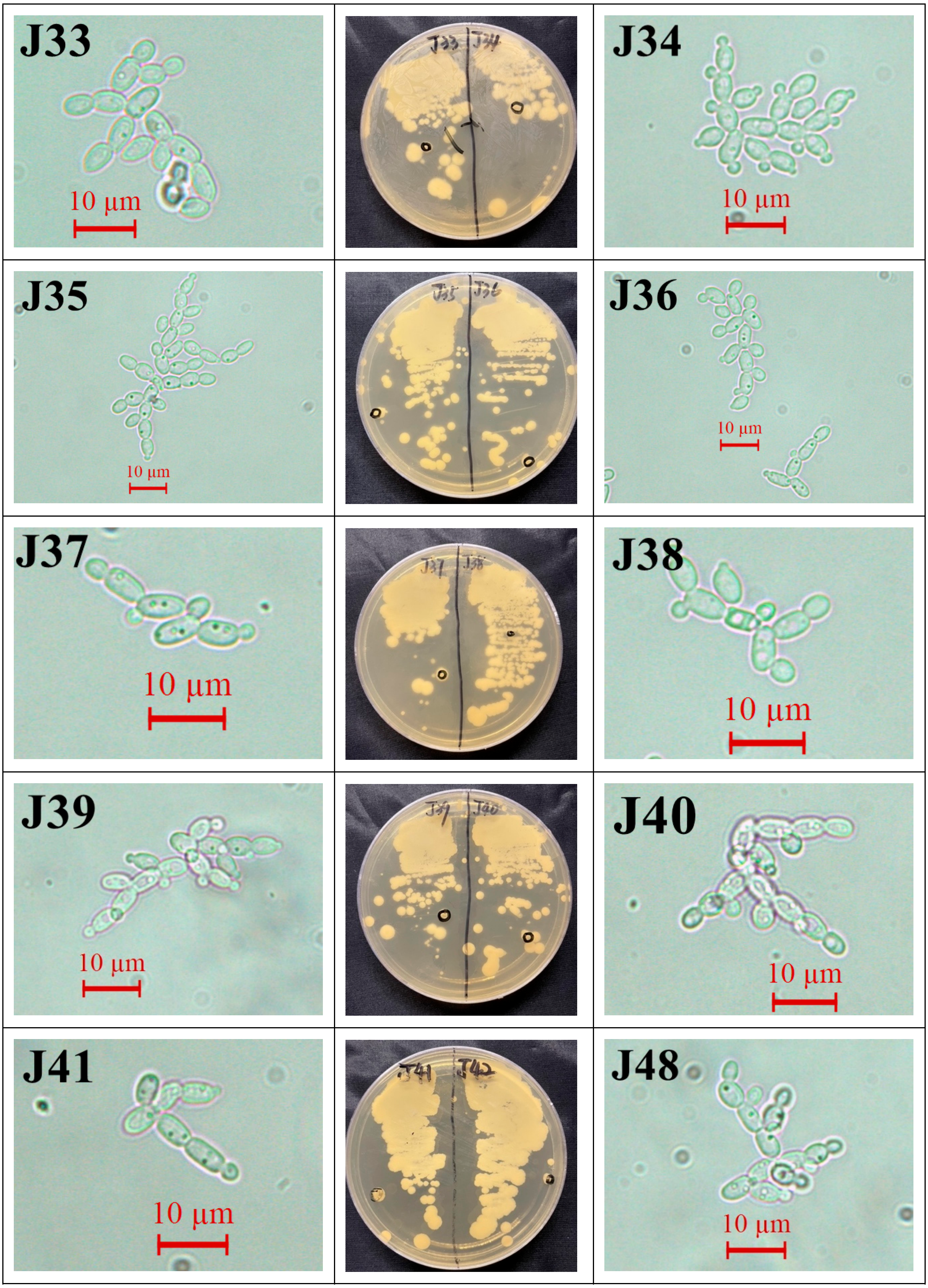

J33
10 µm
J34
10 µm
J35
10 µm
J36
10 µm
J37
10 µm
J38
10 µm
J39
10 µm
J40
10 µm
J41
10 µm
J48
10 µm

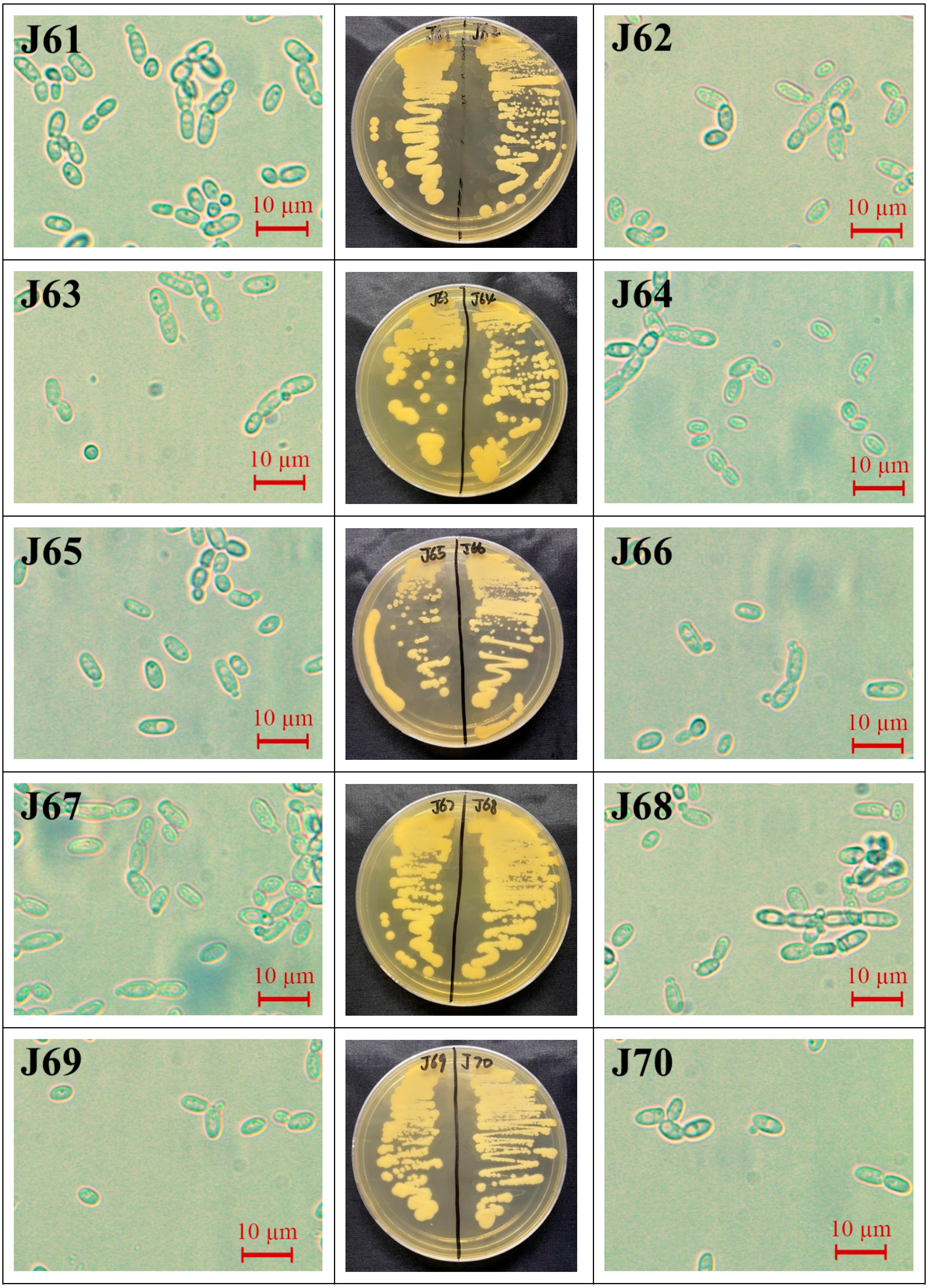
J61
10 µm
J62
10 µm
J63
10 µm
J64
10 µm
J65
10 µm
J66
10 µm
J67
10 µm
J68
10 µm
J69
10 µm
J70
10 µm

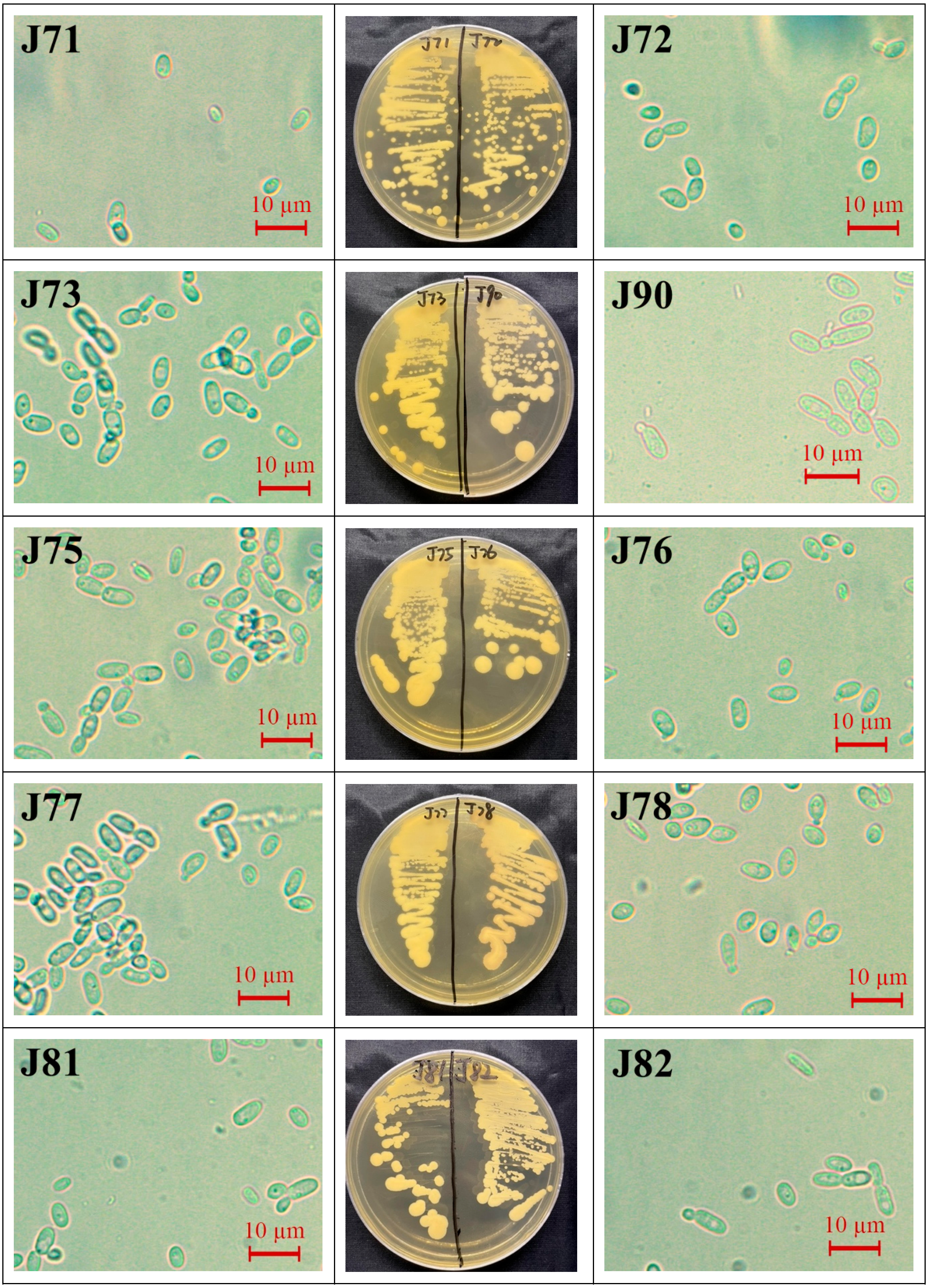
J71
10 μm
J72
10 μm
J73
10 μm
J90
10 μm
J75
10 μm
J76
10 μm
J77
10 μm
J78
10 μm
J81
10 μm
J82
10 μm

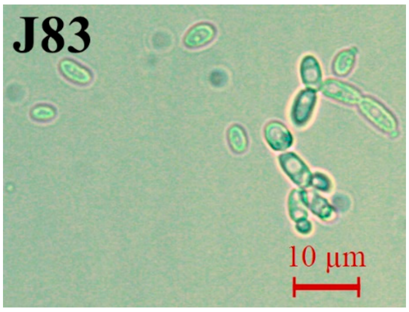
J83
10 µm

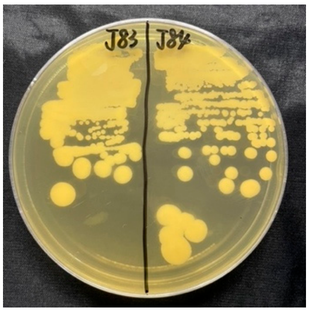
J83
J84

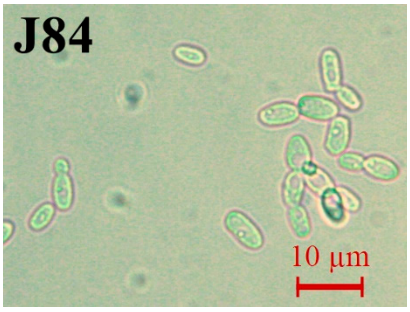
J84
10 µm

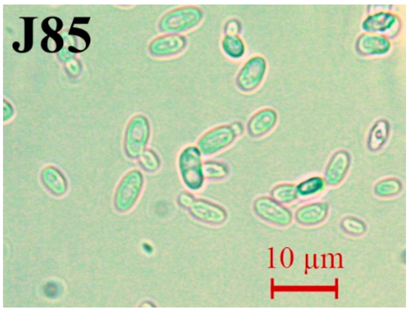
J85
10 µm

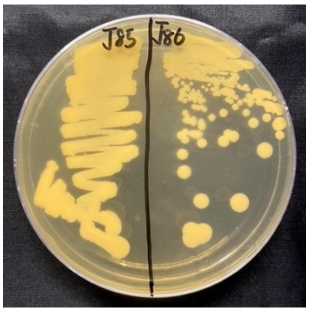
J85
J86

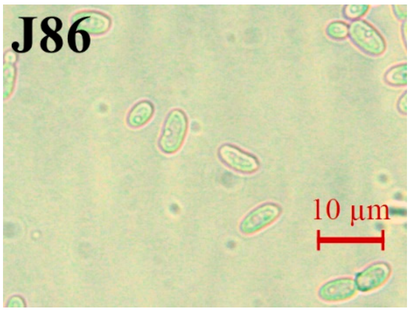
J86
10 µm

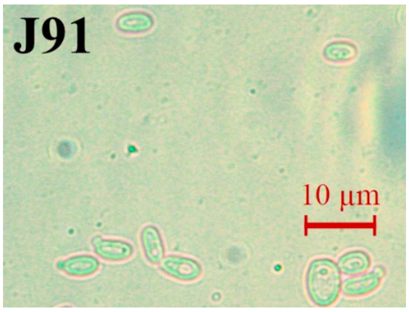
J91
10 µm

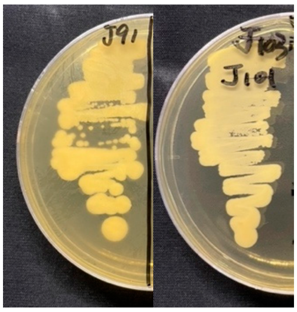
J91
J101

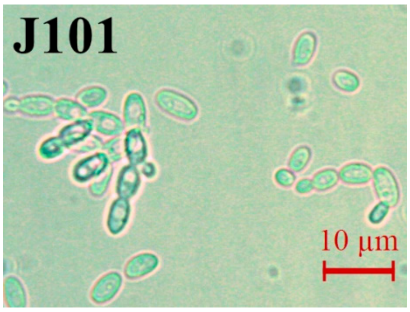
J101
10 µm

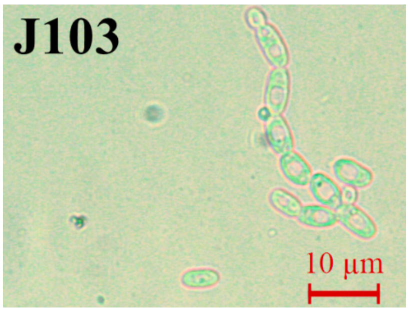
J103
10 µm

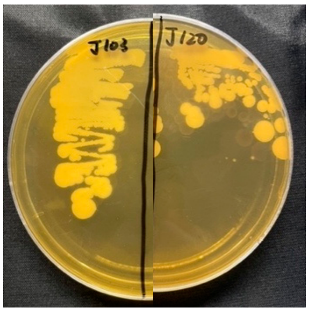
J103
J120

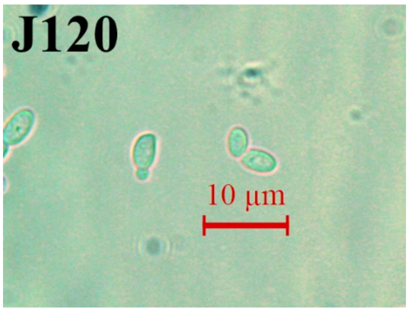
J120
10 µm

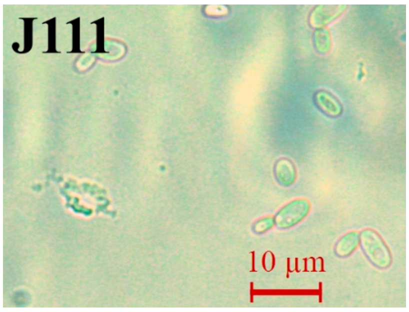
J111
10 µm

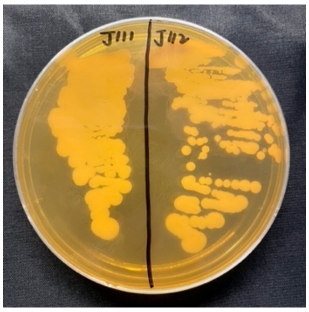
J111
J112

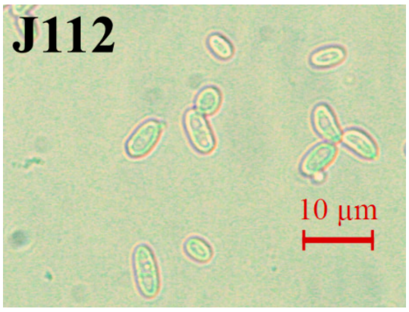
J112
10 µm

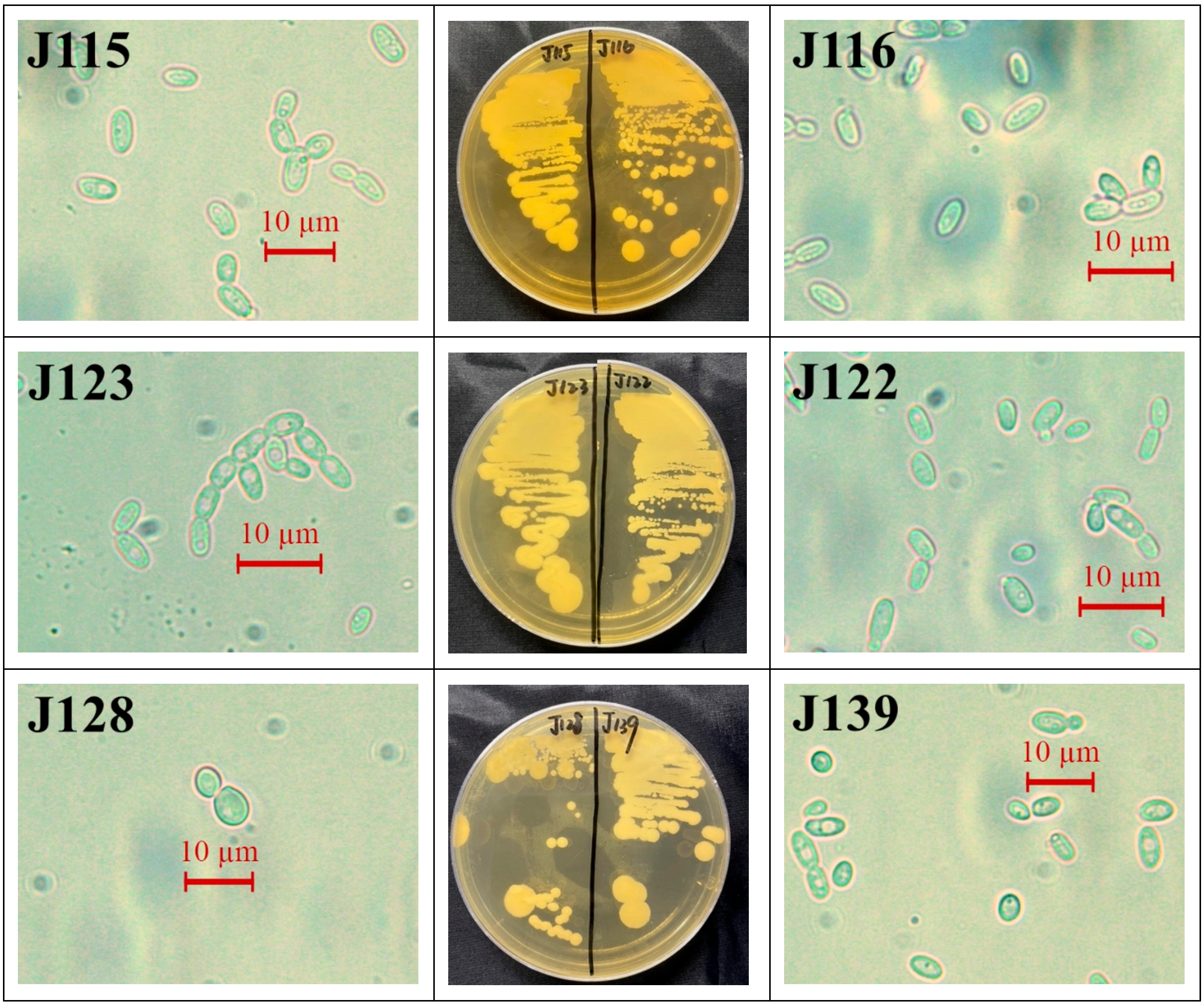

Figure S2 Results of colony morphological analysis and strain morphological analysis.

Table S1 The EC concentration of Chinese Baijiu after treated with immobilized J1, J1, immobilized J116, J116 and chitosan at 4 h, 8 h, 12 h and 24 h.

System	Time	Concentration of EC (μg/L)	Degradation Rate (%)
Baijiu	0	253.03 ± 9.89	/
Immobilized J1	4	242.87 ± 1.14	4.01
	8	203.90 ± 2.07	19.42
	12	173.10 ± 1.36	32.59
	24	146.07 ± 1.67	42.27
Immobilized J116	4	218.64 ± 6.97	13.59
	8	210.36 ± 5.12	14.56
	12	216.20 ± 9.27	16.86
	24	182.42 ± 5.05	27.91
J1	24	200.80 ± 4.17	20.64
J116	24	205.11 ± 2.29	18.93
